# Cytotoxicity of temperature-responsive cationic diblock copolymers in human cancer and non-cancer cells lines

**DOI:** 10.1080/07853890.2021.1896101

**Published:** 2021-09-28

**Authors:** Susana Bandarra, Paulo Mascarenhas, Isabel Barahona, Bo Nyström, Maria Teresa Calejo

**Affiliations:** a Centro de Investigação Interdisciplinar Egas Moniz (CiiEM), Egas Moniz Cooperativa de Ensino Superior, Caparica, Portugal; b Department of Chemistry, University of Oslo, Oslo, Norway; c BioMediTech, Faculty of Medicine and Health Technology, Tampere University, Tampere, Finland

## Abstract

**Introduction:**

Thermoresponsive polymers have been widely investigated in modern drug delivery and gene delivery systems, due to their potential to load and release the payload by simply modifying local temperature [1]. Previous work by some of us has shown that the thermoresponsive copolymers poly(*N*-isopropylacrylamide)*_n_*-*block*-poly((3-acrylamidopropyl) trimethylammonium chloride)*_m_* (PNIPAAM*_n_*-b-PAMPTMA(+)*_m_*) can efficiently deliver DNA and siRNA to HeLa [2] and HT1080 cells [3]. The transfection efficiency and cytotoxicity were highly dependent on the length and ratio of the two blocks (*n*:*m*) [2,3]. In anticancer therapy, such polymers may be able to deliver drugs precisely at the tumour site by local and transient thermal treatment [1]. Having this in mind, in this work, we took the first step towards expanding the scope of use of the PNIPAAM*_n_*-b-PAMPTMA(+)*_m_* copolymers by investigating their cytotoxicity in human cancer cell lines and in a normal breast cell line.

**Materials and methods:**

The copolymers (*n:m* ratio 48:6, 48:10 and 65:20) were dissolved in varying concentrations in cell culture medium, as described before [2] and were incubated at 37 °C with each of the tested cell lines for 6 h. After that, the medium was replaced, and the cells were incubated for 48 h/37 °C. The viability of cells was measured by the MTT cytotoxicity assay. Untreated cells were used as negative control (representing 100% viability). *Cell lines:* HeLa (cervical cancer), HCC1806 (breast cancer) and MCF10A (non-tumorigenic breast epithelial cell line).

**Results:**

In this preliminary study the cytotoxicity of the PNIPAAM*_n_*-*b*-PAMPTMA*_m_*(+) copolymers was shown to be dependent on the polymer concentration, *n:m* ratio and cell line. MCF10A cells were very tolerant to the presence of the copolymer 48:6, even at the highest concentration (Figure 1), followed by HeLa and HCC1806. **Discussion and conclusions:** The low toxicity shown particularly for the non-tumorigenic cell line but higher toxicity towards the cancer cell lines makes the tested copolymers highly appealing as drug carriers in anti-cancer therapy.

**Figure 1.**
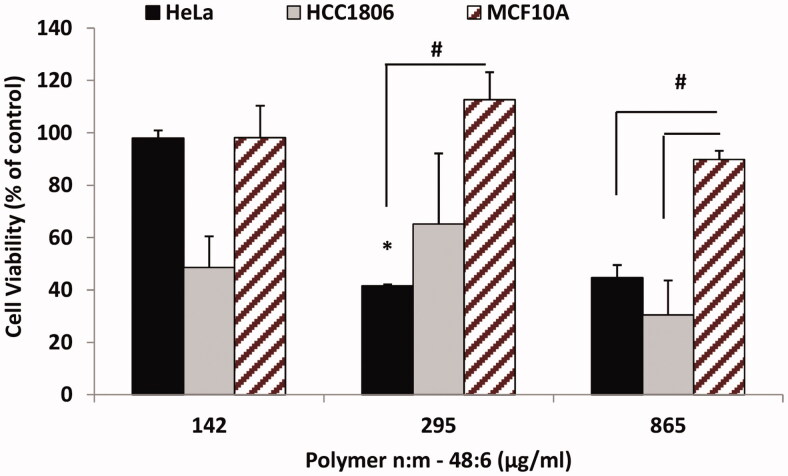
Cell viability in the presence of the 48:6 (n:m) copolymer. Mean ± SD. Comparisons were made at the same cell line at the immediately precedent concentration (*p < 0.05) and for the same concentration between lines (#*p* < 0.05).
